# The Dysfunction is in the Details: Neurovascular Changes in COVID-19

**DOI:** 10.1017/cjn.2020.150

**Published:** 2020-07-15

**Authors:** Houman Khosravani

**Affiliations:** Neurology Quality and Innovation Laboratory (NQIL), Division of Neurology, Department of Medicine, Sunnybrook Health Sciences Centre, University of Toronto, Toronto, Canada

**Keywords:** Stroke, COVID-19, Vasculopathy

Human coronaviruses (HCoVs) can cause a spectrum of disease as indolent as the common cold (described in the 1960s) to severe disease. The dominant phenotype of severe acute respiratory syndrome (SARS) is shared among SARS-CoV-1, Middle East respiratory syndrome (MERS), and now SARS-CoV-2; this latter novel virus shares about 80% of its genomic sequence with SARS-CoV-1. CoVs are enveloped viruses that have single-stranded positive-sense genomes containing approximately 25–32 kilobase pairs as compared to our genome of approximately 3 billion base pairs. In total, seven CoV strains can infect humans including: HCoV-229E (229E), HCoV-OC43 (OC43), SARS-CoV-1, MERS-CoV, HCoV-NL63 (NL63), HCoV-HKU1 (HKU1), and SARS-CoV-2.^1^ Several of these viruses are neurotropic including HCoV-OC43 (in vitro infection of neuronal cultures, and association with multiple sclerosis in CNS tissue in humans), HCoV-229 E, and there is some evidence that SARS-CoV-1 was present in CSF and brain tissues.^2^ It is not fully understood if SARS-CoV-2 is directly neurotropic but emerging data suggest a spectrum of endothelial dysfunction and vaculopathy including endothelitis, and possibly vasculitis. Endothelial cell infection has been described in different organs, including the kidney, liver, heart, and lungs.^3^

Matos et al. describe a case of a young patient, diagnosed with COVID-19, with associated strokes and vasculopathy.^4^ Although large-vessel occlusion (LVO) stroke has been described recently,^5^ it is yet unclear if this is due to other factors such as disseminated viral sepsis rather than a specific pathophysiologic mechanism. Nonetheless, LVO appears to be an uncommon presentation. It is plausible that COVID-19 can exacerbate, through systemic illness, known cerebrovascular risk factors such as atrial fibrillation, and furthermore it is not known if the procoagulable state seen in COVID-19 can directly or secondarily cause strokes.

SARS-CoV-2 enters the cell via its spike protein, being primed by the serine protease TMPRSS2, and interacting with the ACE-2 receptor.^6^ ^,^ ^7^ As more is learned about these specific molecular interactions, in addition to the discovery of others, the relative differences in expression and function of transmembrane proteins involved may vary among microvascular and macrovascular beds. It is these heterogeneities that may lead to a spectrum of neurovascular-based disease manifestations. Given endothelial involvement, it is very plausible that the blood–brain barrier is compromised in COVID-19, which can also secondarily occur due to severe systemic inflammation. Novel molecular targets also open a unique window of opportunity for precision therapeutics. Targets may also be different with differing stages of the infection. Furthermore, some of the consequences of COVID-19 may have lasting neurologic side effects and this is a topic undergoing active investigation.

There are laboratory features of COVID-19 coagulopathy as well that have recently been described, including platelet activation in intensive care unit (ICU) and non-ICU patients. Changes in von Willebrand factor and soluble thrombomodulin can impair activated protein C generation that are felt to promote procoagulation and proinflammatory changes.^8^ These findings highlight endotheliopathy and potential mechanisms of COVID-19-associated vasculopathy.

Several questions remain: does SARS-CoV-2 cause direct endothelial dysfunction due to infection of endothelial cells, compromising the neurovascular unit and the blood–brain barrier or is the dysfunction a consequence of systemic inflammation and cascades related to that pathophysiology – the answer may be both. There is evidence in vitro that SARS-CoV-2 is able to infect human blood vessel organoids.^9^ Another key question is the relation between endothelial dysfunction and a procoagulation state, which may vary between organs, disease severity, and time course. It is also unclear if the pathophysiology is maintained or rather how it is modulated by the degree of cytokine pathways activated, which appear to track a spectrum of disease severity.

Future studies are required to further define the mechanisms involved in endothelial dysfunction due to SARS-CoV-2 in pulmonary, extra-pulmonary, and CNS tissues. Similarly, further investigation is needed with regard to neurotropism.
